# From SNP co-association to RNA co-expression: Novel insights into gene networks for intramuscular fatty acid composition in porcine

**DOI:** 10.1186/1471-2164-15-232

**Published:** 2014-03-26

**Authors:** Yuliaxis Ramayo-Caldas, Maria Ballester, Marina RS Fortes, Anna Esteve-Codina, Anna Castelló, Jose L Noguera, Ana I Fernández, Miguel Pérez-Enciso, Antonio Reverter, Josep M Folch

**Affiliations:** 1 Centre de Recerca en Agrigenòmica (CRAG), Consorci CSIC-IRTA-UAB-UB, Campus UAB, Bellaterra 08193, Spain; 2 Departament de Ciencia Animal i dels Aliments, Facultat de Veterinaria, Universitat Autonoma de Barcelona, Bellaterra 08193, Spain; 3 The University of Queensland, Queensland Alliance for Agriculture and Food Innovation, Center for Animal Science, Gatton, Queensland 4343, Australia; 4 IRTA, Genètica i Millora Animal, Lleida 25198, Spain; 5 INIA, Mejora Genética Animal, Madrid 28040, Spain; 6 Institució Catalana de Recerca i Estudis Avançats, ICREA, Barcelona, Spain; 7 Commonwealth Scientific and Industrial Research Organisation, Division of Animal, Food and Health Sciences, Brisbane, Queensland 4067, Australias

**Keywords:** Pig, Fatty acid, Gene network, Transcription factor, Co-association, Co-expression

## Abstract

**Background:**

Fatty acids (FA) play a critical role in energy homeostasis and metabolic diseases; in the context of livestock species, their profile also impacts on meat quality for healthy human consumption. Molecular pathways controlling lipid metabolism are highly interconnected and are not fully understood. Elucidating these molecular processes will aid technological development towards improvement of pork meat quality and increased knowledge of FA metabolism, underpinning metabolic diseases in humans.

**Results:**

The results from genome-wide association studies (GWAS) across 15 phenotypes were subjected to an Association Weight Matrix (AWM) approach to predict a network of 1,096 genes related to intramuscular FA composition in pigs. To identify the key regulators of FA metabolism, we focused on the minimal set of transcription factors (TF) that the explored the majority of the network topology. Pathway and network analyses pointed towards a trio of TF as key regulators of FA metabolism: *NCOA2, FHL2* and *EP300*. Promoter sequence analyses confirmed that these TF have binding sites for some well-know regulators of lipid and carbohydrate metabolism. For the first time in a non-model species, some of the co-associations observed at the genetic level were validated through co-expression at the transcriptomic level based on real-time PCR of 40 genes in adipose tissue, and a further 55 genes in liver. In particular, liver expression of *NCOA2* and *EP300* differed between pig breeds (Iberian and Landrace) extreme in terms of fat deposition. Highly clustered co-expression networks in both liver and adipose tissues were observed. *EP300* and *NCOA2* showed centrality parameters above average in the both networks. Over all genes, co-expression analyses confirmed 28.9% of the AWM predicted gene-gene interactions in liver and 33.0% in adipose tissue. The magnitude of this validation varied across genes, with up to 60.8% of the connections of *NCOA2* in adipose tissue being validated via co-expression.

**Conclusions:**

Our results recapitulate the known transcriptional regulation of FA metabolism, predict gene interactions that can be experimentally validated, and suggest that genetic variants mapped to *EP300, FHL2,* and *NCOA2* modulate lipid metabolism and control energy homeostasis in pigs.

## Background

Fatty acids (FA) are a major energy source and important constituents of cell membranes, playing a relevant role as cellular signaling molecules in various metabolic pathways, including metabolic diseases [1]. Environmental and genetic effects determining FA composition in pigs have been the subject of many studies. Supporting a genetic influence on FA composition moderate to high heritability estimates have been reported [2,3]. However, the molecular process controlling FA composition and metabolism is far from being fully understood. Technological, nutritional and organoleptic properties of pork meat quality are highly dependent on lipid content and FA composition [4-6]. Thus, elucidating this molecular process could aid improve meat quality for healthy human consumption and increase knowledge of FA metabolism, underpinning metabolic diseases. Pigs are important models for metabolic diseases such as obesity, type II diabetes (T2D) and atherosclerosis [7-10].

Molecular pathways controlling lipid metabolism are highly interconnected. Also, they interact with other related pathways, such as carbohydrate metabolism and energy homeostasis pathways. Together, these pathways and its interactions constitute an essential metabolic network for homeostatic control and normal organism development [11]. In this context, a system biology approach focused on the connections and functional interactions between genes that underpin these metabolic pathways is an attractive alternative to the classical “single-gene-single-trait” approach found in most genome-wide association studies (GWAS) using single nucleotide polymorphisms (SNP).

The main goal of this study was to employ a previously described system biology approach termed Association Weight Matrix (AWM) [12] and, based on a SNP-to-SNP co-association evidence, infer a gene network for intramuscular (IMF) FA composition in pigs. This multi-trait approach was applied to data from 15 phenotypes related to FA composition and metabolism from an Iberian x Landrace intercross. Iberian pigs are a local Mediterranean breed extreme for obesity and appetite [13], whereas Landrace is a lean international breed. The analysis of the predicted gene network revealed key transcription factors that are network hubs and would be critical to determining meat quality, FA composition and controlling energy homeostasis. Finally, we experimentally validated some of the AWM network predictions using real-time PCR gene co-expression analyses in adipose and liver tissues.

## Results

**Figure 1:**
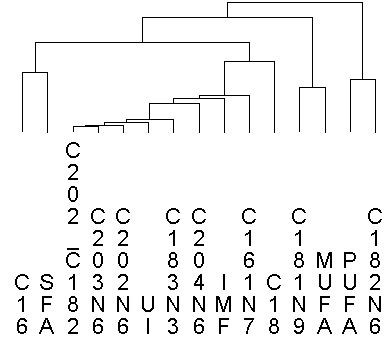
**Hierarchical cluster analysis of the 15 phenotypes analyzed in this study.** Palmitic acid (C16), Stearic acid (C18), Palmitoleic acid (C161N7), Oleic acid (C181N9), Linoleic acid (C182N6), α-Linolenic acid (C183N3), Eicosadienoic acid (C202N6), Eicosatrienoic acid (C202N6), Arachidonic acid (C204N6), Saturated FA (SFA), Monounsaturated FA (MUFA), Polyunsaturated FA (PUFA), Unsaturated indices (UI), Elongase activity (C202|C182), Percentage intramuscular fat (IMF).

**Figure 2:**
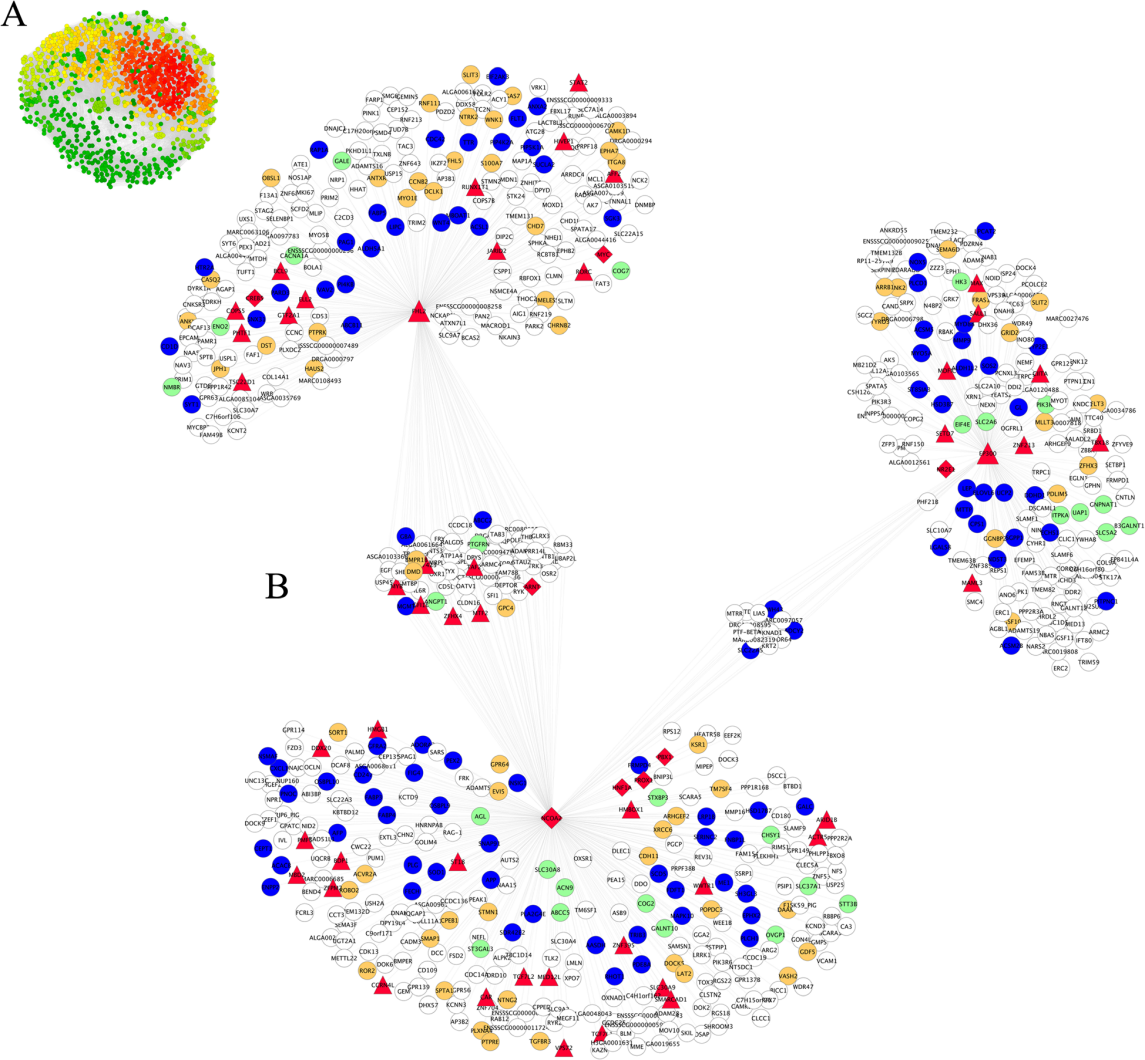
**Co-association network based on the AWM approach. (A)** Entire network with 1,096 nodes (i.e., genes or SNPs) and 111,198 interactions. The color spectrum ranges from green to red for low and high density, respectively. **(B)** Subset of the network showing the best trio of transcription factors: *NCOA2*, *EP300* and *FHL2*. Node color corresponding with the functional classification of the *in-silico* predicted target gene as follows: TF (red), lipid metabolism process (blue), carbohydrate metabolisms (green), development process (orange) and finally, white nodes represent genes with others functional classification. Node shape indicates classification as: diamond (TF involved in lipid metabolism), triangle (TF), ellipse (other genes).

Genotyping data from 48,119 SNPs in 144 backcross pigs (25% Iberian × 75% Landrace) was employed for GWAS of fatty acid related traits in the *Longissimus dorsi* muscle. For all 15 phenotypes, estimated SNP additive effects were standardized (z-scores) by subtracting the mean and dividing by the phenotype-specific standard deviation. After applying a series of selection criteria (see Methods), a total of 1,096 SNPs were retained to build the AWM matrix. Correlations between phenotypes were calculated using AWM columns (standardized SNP effects across traits) and were visualized as a hierarchical tree cluster, in which strong positive and negative correlations are displayed as proximity and distance, respectively (Figure 1). The observed cluster distribution is in concordance with the physiological similarities and relationships among FA. Hence, palmitic acid with saturated FA (SFA), oleic with monounsaturated FA (MUFA), and linoleic with polyunsaturated FA (PUFA) cluster together (Figure 1). Linoleic acid and PUFA are clearly differentiated from other FAs. This result can be explained by the inability of mammals to synthesize linoleic and α-linoleic FAs, which must be provided by the diet. Gene interactions were predicted using pair-wise correlation analysis of the SNP effects across pair-wise rows of the AWM. Hence, the AWM predicted gene interactions based on significant co-association between SNPs. In the network, every node represents a gene (or SNP), whereas every edge connecting two nodes represents a significant interaction. In total, 111,198 significant edges (or 18.5% of all the possible edges) between the 1,096 nodes were identified as significant by the PCIT algorithm [14] (Figure 2A). For every node we computed the total number of connections based on significant interactions. Table 1 lists the ten most connected nodes and Additional file 1: Table S1 their positional concordance with fat-related QTL deposited in the Pig QTL Database.

**Table 1:** Description of the ten most connected nodes in the co-association network

**SNP/Gene**	**Illumina Chip SNP**	**Associated Traits**	**Connections**	**Consequence**
ALGA0061664	ALGA0061664	3	376	Intergenic variant
SLC30A9	H3GA0024739	1	373	Intronic variant
SEMA3F	DIAS0001129	3	370	Intronic variant
ARHGEF2	ASGA0021047	3	368	Downstream variant
NTRK3	MARC0045253	3	367	Intronic variant
ZFHX4	ALGA0025325	1	366	Intronic variant
SLC22A3	ALGA0117149	6	365	Intronic variant
ARMC4	ALGA0059185	4	356	Intronic variant
C9orf171	ASGA0008154	6	355	Intronic variant
PPP2R2A	DIAS0004697	5	353	Splice region variant

Gene ontology (GO) and pathway enrichment analyses were performed to gain insight into the predicted gene network. Overrepresented GO terms in the network included: “Cellular component organization” (*P* = 4.02 × 10^-6^, FDR = 3.95 × 10^-2^), “Cellular component organization or biogenesis” (*P* = 7.34 × 10^-6^, FDR = 3.6 × 10^-2^), “Cell projection morphogenesis” (*P* = 9.59 × 10^-5^, FDR = 9.42 × 10^-2^), “Fatty acid metabolic process” (*P* = 5.89 × 10^-4^, FDR = 1.03 × 10^-2^), “Glycerolipid metabolic process” (*P* = 1.2371 × 10^-3^, FDR = 1.66 × 10^-2^), “Sphingolipid metabolic process” (*P* = 7.45 × 10^-4^, FDR = 1.16 × 10^-2^) and “Unsaturated fatty acid biosynthetic process” (*P* = 2.13 × 10^-3^, FDR = 2.27 × 10^-2^). Additional file 2: Table S2 provides the full list of overrepresented GO terms. Pathway analyses revealed an enrichment for “Regulation of actin cytoskeleton (hsa04810)”, “Focal adhesion (hsa04510)”, “Pathways in cancer (hsa05200)”, “Chemokine signalling pathway (hsa04062)”, “Phosphatidylinositol signalling system (hsa04070)” and “Inositol phosphate metabolism (hsa00562)” (Additional file 3: Table S3).

To identify potential regulators of the above-mentioned pathways and GO categories, we focused on TF found in the gene network. We applied an information lossless approach that explored the 64,824 possible trios among the available 74 TF (see Methods and Additional file 4: Table S4 for complete list of TF) and identified the TF trio that spanned most of the network topology with minimum redundancy. These three TF were: *Nuclear receptor coactivator 2* (*NCOA2*, alias *TIF2*), *E1A binding protein p300* (*EP300*, alias *p300*) and *four and a half LIM domains 2* (*FHL2*, alias *SLIM-3*). Interestingly, the promoter region of these TF contain binding sites for some well-known TF that are considered as important regulators of lipid and carbohydrate metabolism such as: *SREBP-1, PPARG, PPAR-α, HNF1A, HNF4-α, ER-α* and *GR-α*. In the predicted network, a total of 730 genes show co-association with the three key TF (Figure 2B). A detailed examination of the most representative pathways related to these 730 predicted target genes showed a significant overrepresentation for “HIF-1 signaling pathway (hsa04066)”, “Acute myeloid leukemia (hsa05221)”, “Colorectal cancer (hsa05210)”, “Renal cell carcinoma (hsa05211)” and “Type II diabetes mellitus (hsa04930)” (Additional file 5: Figure S1). Admittedly, some of the above-mentioned GO terms and pathways could have been expected from a network predicted from GWAS of FA-related phenotypes and this gives confidence in the reliability of the results. Others, however, were unexpected and might lead to new insights on FA physiology.

### Experimental validation: From co-association to co-expression analysis in liver and adipose tissues

**Figure 3:**
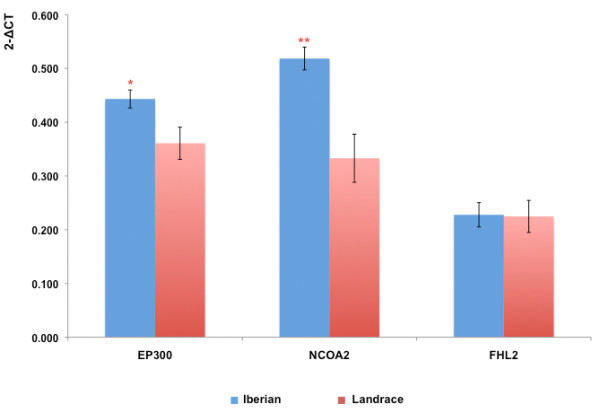
Results of the liver differential expression analysis comparing the best TF trio in the Iberian and Landrace breeds.

The expression of the three TF across *Longissimus dorsi* muscle (LD), adipose and liver tissues was explored. In concordance with previous results suggesting that highly connected TF are in general broadly expressed across tissues [15], the three TF were expressed across all the studied tissues. Further, a comparison between Iberian and Landrace pig breeds revealed significant increase fold changes (FC) in the liver of Iberian pigs for the expression of *NCOA2* (FC = 1.56, *P* < 0.01) and *EP300* (FC = 1.23, *P* < 0.05) (Figure 3).

The expression patterns of 43 genes in liver and 40 genes in adipose tissue were successfully measured across 55 backcross animals. In liver, the expression data of twelve additional genes were also included in the co-expression analysis (see Methods). Co-expression analysis revealed highly connected networks in both liver and adipose tissue, suggesting strong functional interconnections among the studied genes. Topology of liver co-expression network showed 55 nodes connected by 425 edges (Additional file 6: Figure S2A) and in adipose tissue 40 nodes and 261 edges were observed (Additional file 6: Figure S2B). Network parameters such as average degree (Deg) and average distance (AvD_G_) were slightly higher in liver co-expression network compared to adipose tissue network (Deg_Liver_ = 15.45 AvD_G_ = 1.81 vs Deg_Adipose_ = 13.05 and AvD_G_ = 1.75). Based on network centrality, the relevance of individual genes differs within each network. For example, topological properties of the liver co-expression network suggest an important role for *ARNT* in the regulation of hepatic lipogenic and glucoconeogenesis activity, and these findings agree with published results [16,17]. It should be noted that *BCL9* showed the highest centrality value in the liver co-expression network (Additional file 6: Figure S2A). In addition, degree analysis showed that *BCL9*, *EP300, PBX1, SIRT1, PIP5K1A* and *ARNT* were the most central genes in the liver co-expression network. However, in the adipose co-expression network, degree analysis suggested that *ANK2, NCOA2, SIRT1, EIF4E, HMBOX1* are the most central genes (Additional file 6: Figure S2B). When analysing a sub-network of the liver co-expression network, formed only by the same 40 genes included in the adipose co-expression network, five genes (*BCL9, EP300, PBX1, PIP5K1A, and SIRT1*) were still the most central genes and this finding underscores their relevant role in the function and structure of the liver co-expression network.

**Figure 4:**
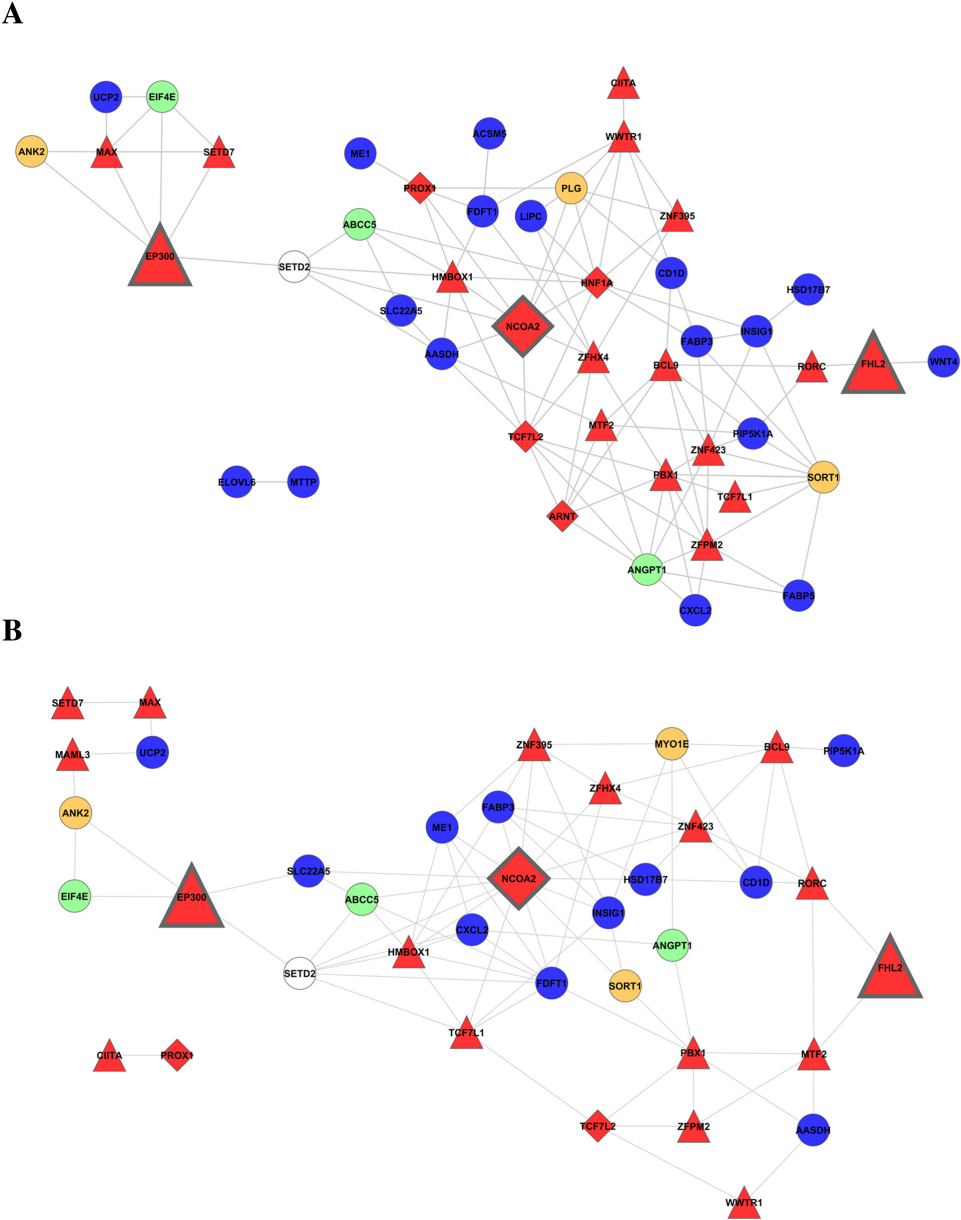
**Connections from the co-association network that were confirmed by the co-expression network in liver (A) and adipose (B) tissue.** Nodes color relate to the functional classification of genes as follows: TF (red nodes), lipid metabolism (blue nodes), carbohydrate metabolism (green), development process (orange) and white nodes represent genes with others functional classification. The size of the nodes corresponding to the best trio of transcription factors (*NCOA2*, *EP300* and *FHL2*) has been enlarged to facilitate their location.

Beyond the study of the topological properties of the liver and adipose tissue co-expression networks, we were concerned with those, if any gene interactions predicted via SNP co-association were corroborated through co-expression analyses. In line with recent results in yeast [18], we observed that interacting loci could jointly regulate the co-expression patterns of pairs of genes. For the first time in a not model species, co-expression analyses confirmed gene-gene interactions predicted based on SNP co-association. However, the magnitude of this validation varied in a tissue-specific manner. For instance, with respect to the liver module formed by 48 AWM nodes and 359 edges (based on co-association analysis) we observed that 28.9% (104/359) of the predicted gene-gene interactions were validated by the co-expression results. Whereas in the adipose tissue, the observed percentage of the AWM validated interactions was slightly higher representing 33.0% of the possible combinations (Figure 4B). When we limited this comparison to the intersecting 39 genes included in both co-expression networks, the proportion of the AWM gene-gene interactions validated in liver (29.5%) was still lower than in adipose tissue (33.0%). Comparing both networks, we observed that approximately 35.7% (or 30 out of 84) of the interactions validated in the adipose tissue were also validated in the liver co-expression analysis (Additional file 7: Table S5). Interestingly, these always co-associated and co-expressed genes belong to biological processes related to lipid metabolism including: Negative Regulation of Fat Cell Differentiation (*INSIG1, TCF7L2, ZFPM2*), Androgen Receptor Signalling Pathway (*EP300, FHL2, NCOA2*), Response to Hormone Stimulus (*ABCC5, ANGPT1, FABP3, EP300, SORT1, FHL2*) and Lipid Metabolic Process (*PBX1, INSIG1, FABP3, FDFT1, PIP5K1A, MAX, AASDH*).

When we focused on the best TF trio, we observed that 60.8% (or 14 out of 23) of the interactions of *NCOA2* predicted by the AWM co-association network were corroborated in the co-expression network of the adipose tissue. This percentage dropped to 34.6% (or 9 out of 26) in the co-expression network of the liver tissue. For *EP300*, 44.4% (or 4 out of 9) of the AWM predicted interactions were observed in the adipose co-expression network and 41.6% (5 out of 12) in the liver co-expression network. Finally, for *FHL2* we observed the lowest percentage of validated interactions: 20.0% (or 2 out of 10) in adipose tissue and 14.3% (2 out of 14) in liver (Table 2).

**Table 2:** Concordance validation between the co-association and the co-expression networks for the best TF trio and in adipose and liver tissues

**Tissue**	**TF**	**Connections in the AWM co-association network**^**A**^	**Connections in the qPCR co-expression network**^**A**^	**Validated connections**	**% Validation**
Adipose	NCOA2	23	21	14	60.8
	EP300	9	19	4	44.4
	FHL2	10	9	2	20.0
Liver	NCOA2	26	18	9	34.6
	EP300	12	28	5	41.6
	FHL2	14	13	2	14.3

^A^Connections deemed significant according to the PCIT algorithm.

## Discussion

Molecular processes controlling FA metabolism are highly interconnected and linked with related pathways, such as lipid, carbohydrate and energy metabolism. In fact, FA are a major energy source and together with several factors, such as total energy intake, dietary fat/carbohydrate ratio, or glucose and/or insulin concentration, regulate *de novo* lipogenesis [19,20]. As a consequence, it is expected that at the selected threshold (*P* < 0.035) our best trio of TF (*NCOA2, EP300, FHL2*) show co-association with a large number of genes and other TF relevant for lipid, carbohydrate and energy metabolism. For instance, 39 of the predicted target genes via SNP co-association (Additional file 8: Table S6) have been recently reported in two large-scale meta-analysis studies for plasma lipids in humans [21,22]. Interestingly, many of these genes, including our TF trio and other FA relevant genes, would have been missed by traditional single-trait GWAS due to the lack of an acceptably significant association level (i.e. *P* > 0.05 after correction for multiple testing). As noted before [12] and confirmed by this study, AWM points to new candidate genes, TF and gene interactions via exploring SNP co-associations across multiple traits beyond the one-dimensional approach for identifying genes affecting single traits. However, results should be interpreted with caution due to the limited sample size used in our study (144 pigs), which reduces the power to identify small effects and may introduce spurious results. Therefore, these TF might regulate other important genes for IMF FA composition not represented in this network and false positive results may be included in the network. However, only the SNPs associated with a large number of phenotypes were included in the AWM analysis and, due the multi-trait nature of the AWM methodology, the probability that the same SNP was associated with several phenotypes by chance is much lower than the probability of being associated with a single phenotype.

In the predicted network, *NCOA2,* a key TF regulating energy homeostasis [20,23] and adipogenesis [24], showed co-association with a total of 326 genes, including relevant TF and genes associated with lipid and carbohydrate metabolisms, such as *PROX1, PBX1, ARNT, MYB, MTF2, TCF7L1, SCD5, ABCC2, INSIG1, ACACB, FABP4, FABP3, ME1, AASDH, ABCC5 and SORT1*. A role for *PROX1* in the control of energy homeostasis has been proposed [25]. Moreover, association of SNPs mapped to *PROX1* and *SLC30A8* with fasting glucose levels and increased risk for T2D has been reported in humans [26]. Both *PROX1* and *SLC30A8*, together with other T2D risk loci (*IL6R, TCF7L2, HNF1A*) and 21 genes reported as associated with plasma lipids in humans [22] were predicted as target genes of *NCOA2* in our study. Co-expression analysis in adipose tissue validated 60.8% of the *NCOA2* co-association target genes, including *INSIG1* (r_co-expression_ = 0.68), *FDFT1* (r_co-expression_ = 0.70), *SETD2* (r_co-expression_ = 0.59) and *ABCC5* (r_co-expression_ = 0.65). In liver, 34.6% of the predicted targets of *NCOA2* were validated, including the above-mentioned *PROX1* (r_co-expression_ = 0.48), *HNF1A* (r_co-expression_ = 0.56) and *TCF7L2* (r_co-expression_ = 0.50). It should be noted that previous studies in pigs show a correlation between *NCOA2* expression (r = 0.605, *P* < 0.01) and IMF content of LD muscle [24]. Also, *NCOA2* was reported as modulating an AWM-network predicted for puberty in cattle [27], which included fat deposition measurements as traits related to puberty. Furthermore, knockout *NCOA2*^-/-^ mice are protected against obesity, showing lean phenotype and decreased expression of genes involved in the uptake and storage of FA [20]. A decreased expression of genes required for FA synthesis in liver tissue of *NCOA2*^-/-^ mice was observed [28]. In agreement with these previous results and the phenotypic difference in fat deposition between Iberian and Landrace breeds, a significant higher activity of *NCOA2* in the liver of Iberian pigs was detected (FC = 1.56, *P* < 0.01) relative to Landrace pigs (Figure 3).

Another TF predicted as critical for FA regulation was *EP300,* which encodes the adenovirus *E1A-associated cellular p300 transcriptional co-activator protein*. It functions as histone acetyltransferase that regulates transcription by chromatin remodelling. Via histone acetyltransferase activity, *EP300* regulates the transcription of liver X receptor (*LXR*) [29]. *EP300* is also required for adipocyte differentiation through the regulation of peroxisome proliferator-activated receptor gamma (*PPARG*) [30]. Remarkably, *EP300* has been reported as transcriptional co-activator of estrogen receptor (*ER*), hepatocyte nuclear factor 4 α (*HNF4-*α), aryl hydrocarbon receptor nuclear translocator (*ARNT*) and hepatocyte Nuclear Factor-1 α (*HNF1A*) [31-33]. All these above-mentioned TF co-regulated by *EP300* (*PPAR*G, *LXR*, *HNF4, HNF1A, ER, ARNT*) influence lipid and carbohydrate metabolisms and have been extensively studied in this context [17,34-41]. Among the 180 AWM-predicted target genes for *EP300*, there are 30 genes known to be involved in lipid metabolism including *ARNT* a member of the HIF-1 pathway. *ARNT* is a relevant TF regulating hepatic gluconeogenesis and lipogenic gene expression [16]. Interestingly, we observed a significant co-expression between *ARNT* and *EP300* (r = 0.61) in the liver network. Additionally, other genes related to carbohydrate and lipid metabolism were predicted as *EP300* AWM-target genes. These included: *ADCY2, MMP9, ECHS1, ARRB1, EIF4E, ANK2, NR2E1, SLC2A6, SLC5A2, LEP, ELOVL6, MTTP, ACSM5, UCP2* and *CYP2E1* (for a full list see Additional file 9: Table S7). Similarly to *NCOA2*, a significant higher expression of *EP300* in the liver of Iberian pigs was detected (FC = 1.23, *P* < 0.05) in comparison with Landrace pigs (Figure 3). Our results, predicting targets for *EP300* and studying their co-expression contributes to the knowledge on lipid and carbohydrate metabolism. It is well known that TF require co-regulators to modify and epigenetically remodel chromatin structure to facilitate the basal transcriptional machinery. *EP300* is a chromatin remodeling gene opening new possibilities to study the roll of epigenetic modifications in the regulation of pork meat quality and the molecular control of energy homeostasis.

The third key TF was *FHL2,* an evolutionarily conserved gene that can interact with an important range of proteins from different functional classes, including receptors, signal transducers, TF and cofactors [42]. *FHL2* plays an important role as molecular transmitter linking various signalling pathways to transcriptional regulation. For instance, *FHL2* is involved in the co-activation of human androgen receptor (*AR*), *ER* and peroxisome proliferator-activated receptor alpha (PPARα) [42-44]. In addition, *FHL2* mediates interaction with β-catenin and promotes myoblast C2C12 differentiation in mice [45]. The gene *B-cell CLL/lymphoma 9 (BCL9)*, an activator of the Wnt/β-catenin [46] and *Wingless-type MMTV integration site family, member 4* (*WNT4*) was among the 251 targets predicted for *FHL2* in our network. The growth factor *WNT4* is a member of the Wnt signaling pathway involved in developmental processes and relevant for gonad development and sex-determination [47]. Liver expression analyses provided supporting evidence for the predicted interaction between *FHL2* and *WNT4*, as a significant co-expression (r = 0.44, P < 0.001) was observed. Other genes and TF associated with development process, lipid and carbohydrate metabolism, such as *FHL5*, *MYO1E, MYB, RORC, JARID2*, *ZFHX4, WNK1, LIPC, CREB5, CDC42, ACSL1, FABP5, ABCB11, FLT1* and *HTR2A* were also predicted as targets of *FHL2* according to the co-association network. *FHL2* was not differentially expressed in the comparison between Iberian and Landrace pigs. Also, *FHL2* showed a proportion of validated interactions in the co-expression analysis (20% adipose tissues and 14.3% in liver) lower than for the other two TF, *NCOA2* and *EP300*. These somewhat less promising results could be a consequence of the tissue-specific activity of *FHL2,* as it has been reported for the co-activation of *AR*[43].

Although, some gene to gene interactions predicted by the AWM approach were not corroborated by the co-expression analysis, the possibility of these interactions occurring in other spatial temporal and/or tissues cannot be ruled out, or indeed manifesting their joint effect through other means than co-expression. TF and their target genes interact in a temporal and tissue dependent manner, so the examination of networks spanning multiple tissues is critical to highlight interactions that could otherwise be unknown from individual tissue analysis [48]. In spite of this tissue/time limitation, two of the three TF from the best trio (*EP300* and *NCOA2*) showed higher than average centrality values in both liver and adipose tissue co-expression networks. Moreover, we observed a significant co-expression between *NCOA2* and *EP300* in the liver network with some other TF considered master regulators of the lipid metabolism. For instance, *NCOA2* was significantly co-expressed with *PPARα* (r = 0.39, P < 0.01), *HNF1A* (r = 0.56, P < 0.001) and *HNF4α* (r = 0.36, P < 0.01), and *EP300* was co-expressed with *PPARD* (r = 0.38, P < 0.01) and *HNF1A* (r = 0.64, P < 0.001) (Additional file 6: Figure S2 A, B). The liver plays a central role in maintaining overall energy balance by controlling lipid and carbohydrate metabolism. In pigs, the liver is the primary site of *de novo* cholesterol synthesis and fatty acid oxidation and, together with adipose tissue, has a crucial role in regulating lipid metabolism [49,50]. All these observations, together with the higher expression of *NCOA2* and *EP300* observed in the liver of the Iberian pigs compared with Landrace pigs, suggest a relevant role of these genes in the hepatic transcriptional regulation of lipid metabolism in pigs.

Overall, our GWAS and network predictions, supported by literature and co-expression analysis in liver and adipose tissue, suggest a co-operative role for the three TF (*NCOA2, EP300, FHL2*) in the transcriptional regulation of IMF, FA composition and the control of energy homeostasis in pigs. We hypothesize that these TF mediate a highly inter-connected regulatory cascade including pathways such as HIF-1, AR, ER and Wnt/β-catenin that seem pivotal for lipid metabolism. The role of these pathways in the transcriptional regulation of lipid metabolism is a subject of intense studies [17,38,39,51-54]. A functional cooperation between the three TF in the modulation of these pathways is evident from our results and supported by literature evidence. For example, according to String database [55,56] (http://string-db.org/), experimental data confirmed that protein-protein interaction exists among, *EP300, NCOA2, FHL2, AR* and *ESR1* (Additional file 10: Figure S3). In addition, *EP300* and *NCOA2* take part on the AR and ER pathways and both, *NCOA2* and *FHL2* are AR co-regulators [43,57,58]. Studying the combined effect of *NCOA2, EP300,* and *FHL2* in the regulation of specific genes will lead to new knowledge related to FA pathways.

The most overrepresented pathway corresponding to the 730 AWM-predicted target genes of the three TF was HIF-1 (Additional file 5: Figure S1). The HIF-1 pathway is central to adaptive regulation of cellular energy metabolism; by regulating the expression of glycolytic enzymes and hepatic lipid metabolism [17,54,59,60]. Our liver co-expression analysis supports previously reported evidence [16] for the relevance of *ARNT* gene (member of HIF pathway) in the hepatic lipogenic gene expression. Additionally, HIF-1α, which is another member of HIF pathway and β-catenin co-ordinately enhance AR transactivation. The interaction between β-catenin and both HIF-1 and AR pathways has been documented [61-63]. Moreover, β-catenin is a ligand-dependent co-activator of AR and a functional cooperation in the synergistic activation of AR-mediated transcription among *EP300*, *FHL2* and β-catenin have been reported [64]. Ours results showing the interactions between the three key TF, recapitulate these pathways interactions that are known mammalian biology, extending its significance to pigs.

## Conclusions

In summary, our results suggest that common genetic variants mapped to (or in *linkage disequilibrium* with) *EP300*, *FHL2* and *NCOA2* together with other candidate genes including *ARNT*, *BCL9, SIRT1, PBX1, PROX1, HNF1A, SLC30A8, TCF7L2* and *ANK2* modulate lipid metabolism and control energy homeostasis in pigs. Furthermore, epistatic predicted interactions between TF and their target genes are likely to contribute to the complex inheritance of FA composition and related polygenic traits (lipid metabolism and energy homeostasis). It is generally accepted that metabolic diseases such as obesity and T2D are linked to disturbance of energy homeostasis or homeostatic imbalance. It should be noted that among the 730 predicted target genes, an overrepresentation of genes from the T2D pathway was observed (Additional file 5: Figure S1). Also, 39 of the 730 genes are known to control plasma lipid content in humans [21,22].

Further studies will be required to elucidate the specific cellular and molecular processes of interaction among the three TF and its target genes that determine FA composition and control energy homeostasis in pigs. The implications of research in this area are broad, ranging from applications from pork meat quality to modeling mammal biology.

## Methods

### Phenotypic traits, animals and genotypes

Data from 144 pigs (25% Iberian × 75% Landrace), representing 26 full-sib families, from backcrossing five F1 males with 26 Landrace sows was utilized. Details about the management conditions and the phenotype information have been previously reported [65-67]. For this study and based on an previous principal components analysis [66] we selected 15 of the total 48 traits representing the most informative phenotypes within the dataset. Nine of the 15 traits were related to IMF fatty acid (FA) composition in LD muscle, seven correspond to indices of FA metabolism and the last one is the IMF percentage (Additional file 11: Table S8). The Porcine SNP60K BeadChip (Illumina) [68] was used to genotype a total 197 pigs, including the 144 phenotyped animals and the founder population. Quality control excluded SNPs with minor allele frequency < 5% and with call rate < 95%. A subset of 48,119 SNPs were retained for subsequent analysis, in addition, previously detected polymorphisms in the *MTTP, FABP4, FABP5,* and *ELOVL6* genes were also tested [67,69,70]. The genomic coordinates of the SNP correspond to the *Sus scrofa* genome sequence assembly (Sscrofa10.2, August 2011) [71] and were annotated using as reference the pig assembly 10.2 [ftp://ftp.ncbi.nlm.nih.gov/genomes/Sus_scrofa/GFF/].

### Ethics statement

Animal care and procedures were performed following national and institutional guidelines for the Good Experimental Practices and approved by the Ethical Committee of the Institution (IRTA- Institut de Recerca i Tecnologia Agroalimentàries).

### Statistical analysis

The GWAS was performed using Qxpak 5.0 software [72]. The additive effect of a SNP on each trait was estimate by mixed model [73,74] following the model:

$$y_{\mathrm{ij}}=\mathrm{X\beta}+\mathrm{Zu}+s_{j,k}+e_{\mathrm{ij}},$$

where: y_ij_ represents the vector of observations from the i^th^ pig at the j^th^ trait ; X is the incidence matrix relating fixed effects in ß with observation in y_ij_; Z is the incidence matrix relating random additive polygenic effects in *u* with observation in y_ij_; s_j,k_ represents the additive association of the k^th^ SNP on the j^th^ trait and e_ij_ is the vector of random residual effects. Fixed effects included in ß were, sex (two levels), batch (five levels) and carcass weight as covariate. Polygenic effects were treated as random and distributed as N(0, **A**σ_u_) where **A** is a numerator of kinship matrix. Then, the allele substitution effect of the i^th^ SNP on the j^th^ trait was z-score standardized and employed to constructing the AWM [12].

An R script, available from the authors, was written to automate the process of building an AWM. Palmitoleic acid (C16:1 (n-7)) was used as the key phenotype and the procedure described by Fortes and colleagues [12] was followed, but we introduced a few modifications, specifically regarding the *P*-value threshold for selecting SNP from GWAS. The *P*-value threshold was chosen by exploring the sensitivity of the data instead of simply accepting the nominal *P* < 0.05. We took advantage of the biological knowledge concerning TF related to the analyzed traits and used it as *a priori* information.

In essence, instead of applying a hard-coded nominal P-value of, for instance, 0.05 or 0.01 or 0.001, we employed a knowledge-based approach to identify the P-value threshold at which the information content, in terms of fatty acid regulation, is maximized. To this effect, we mined the literature and relevant databases to compile a list of 340 TF of which 34 were known to be related to FA metabolism. The distribution of these 34 TF relative to the entire set of 340 was explored at various P-value thresholds. The P-value threshold that maximized the number of FA-related TF was used as the optimal P-value to apply when developing the Association Weight Matrix. Quite importantly, others in the past have employed a knowledge-based approach to identify the critical P-value threshold. More recently, and in the context of GWAS, Yang et al. [75] used an approach similar to ours to find the P-value that maximise the correlation between the proportion of significant SNPs and the heritability across 47 traits.

In detail, the process for choosing the threshold was as follows:

**Figure 5:**
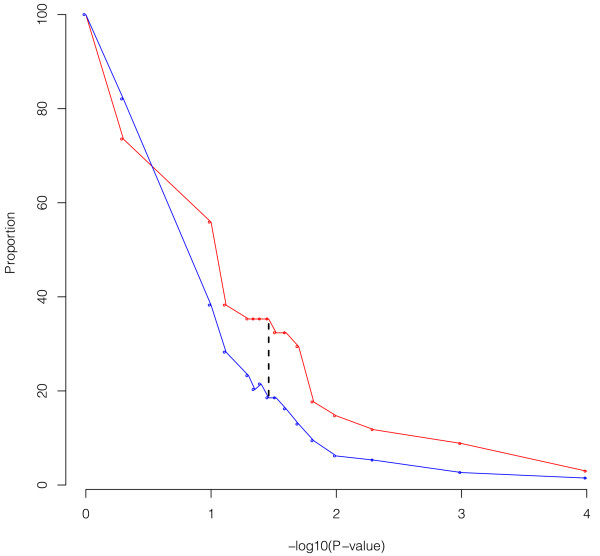
**Sensitivity analysis of the 34 lipid-related TF at different *P*-values (form *P*** **< 1 to *P*** **< 10**^**-4**^**) against the distribution of the total 340 TF included in the dataset.**

**Step1:** A total of 340 TF were located within 2.5 Kb of a SNP and therefore included in the initial dataset. For all these TF included in our dataset, those that are well known key regulators of the lipid metabolism were initially selected.

**Step2:** For each gene, those involved in the lipid metabolism and also reported in the census of human TF by Vaqueriza et al. [76] were included.

**Step3:** The Human Protein Reference Database (HPRD) and the Biomolecular Object Network Databank (BIND) were mined. Then other TF that have been reported to interact with some of the TF retained in the two previous steps were selected. After these first 3 steps, a total of 34 TF were retained (Additional file 12: Table S9).

**Step4:** Subsequently we compare the distribution of the 34 TF at different *P*-values from *P* = 1 to *P* = 10^-4^*versus* the distribution of the total number of TF included in the AWM (340). As a result, we have chosen *P* < 0.035 as the threshold. This specific *P*-value maximizes the difference between both groups of TF (Figure 5), imposing an informed bias towards lipid metabolism to the network.

After defining the threshold of *P* < 0.035, the selection of SNPs for building AWM continued. Those SNPs that were either associated (*P* < 0.035) with palmitoleic acid or with any ≥ 3 traits, and were located either ≤ 2,500 bp to or ≥ 850 kb from the nearest annotated gene (Sscrofa10.2 assembly), were selected to build the AWM matrix. PermutMatrix software [77] was employed to visualize hierarchical clustering of traits (AWM columns) and genes (AWM rows) using Euclidean distance and the Average linkage method. To identify and report gene-gene or gene-SNP interactions we used PCIT algorithm [14]. Cytoscape software [78] was used to visualize the gene network and also to perform overrepresented GO terms analysis, using BiNGO plugin [79]. Node centrality values and network topological parameters were calculated using CentiScaPe plugin [80]. Pathway enrichment analysis were performed using FATIGO tool form BABELOMICS [81,82]. Further, pathways analyses of the 730 predicted target genes (co-associated with the key TF) were performed using ClueGO, Cytoscape plugin [83]. Pathway information was retrieved from the KEGG (http://www.genome.jp/kegg/) and BioCarta (http://www.biocarta.com/) databases. In all cases, the cut-off for considering a significance overrepresentation was established by Benjamini & Hochberg multiple testing correction of the *P*-value (FDR < 0.05) [84].

### Expression and co-expression analysis

In order to provide supporting evidence for the *in-silico* AWM-network predictions we obtained and explored gene expression data by reverse transcription quantitative Real-Time PCR (RT-qPCR). The expression pattern of the 3 Key TF (*NCOA2, EP300, FHL2*) in LD muscle, liver and adipose tissues was tested in two phenotypically divergent breeds for fat deposition traits (Iberian and Landrace which are also the founders of our studied population, five animals per breed). Finally, liver and adipose co-expression analyses of 55 (43 from the present study, and twelve: *ACSM5, APOA2, ARNT, CYP7A1, FABP5, FADS3, HNF4a, LIPC, MTTP, PPARA, PPARD* and *ELOVL6* genes from Ballester et al., 2013 submitted) and 40 genes, respectively, were performed using the PCIT algorithm [14] in 55 backcross animals. Since sex differences in liver transcriptome have been reported [85] only females were considered in the co-expression analyses of both tissues.

From the 55 genes explored in the liver co-expression analysis, 48 were present in the AWM network. The remaining seven were incorporated due to their biological relevance, including three well-know TF related to lipid metabolism (*PPARα, PPARD, HNF4α*) and four genes related to lipid metabolism (*SIRT1, FADS3, APOA2, CYP7A1*). Similarly, from the 40 genes employed in the adipose co-expression analysis, 39 were present in the AWM network. The one gene out, *SIRT,* was also included due to its relevant controlling lipolysis [86,87] and promoting fat mobilization in white adipose tissue [88].

Total RNA was obtained from liver, muscle and adipose tissues using the RiboPure kit (Ambion), following the manufacturer’s recommendations. RNA was quantified using the NanoDrop ND-1000 spectrophotometer (NanoDrop products) and the RNA integrity was assessed by Agilent Bioanalyzer-2100 (Agilent Technologies). Approximately, one microgram of total RNA was reverse-transcribed into cDNA using the High-Capacity cDNA Reverse Transcription kit (Applied Biosystems) in 20 μl of reactions, following the manufacturer’s instructions.

To analyze the expression pattern of the 3 key transcription factors, an ABI PRISM 7900 Sequence Detection System (Applied Biosystems) in combination with FastStart Universal Sybr green master (Rox; Roche Applied Science) was used. PCR amplifications were performed in a total reaction volume of 20 μl containing 5 μl of cDNA diluted 1:25. All primers were used at 300 nM. The thermal cycle was 10 min at 95°C, 40 cycles of 15 s at 95°C and 1 min at 60°C. A dissociation curve was drawn for each primer pair to assess the specificity of the amplification. Three reference genes (*ACTB, HPRT1, TBP*) frequently used in RT-qPCR experiments were tested as endogenous controls. Using the GeNorm software [89], the *ACTB* and *TBP* genes were selected as the best endogenous controls for all tissues. After ensuring the possibility to use the 2^-ΔΔCT^ method [90], data was analyzed using the RQ manager v1.2.1 and the DataAssist™v3.0 softwares (Applied Biosystems). The 2^-ΔCT^ values were used to compare our data.

The 48.48 microfluidic dynamic array IFC chip (Fluidigm) was used to analyze the expression of 48 genes (44 target genes and 4 reference genes) in liver and adipose tissue of 55 backcross animals belonging to the same population in which the GWAS was performed. Two μl of 1:5 diluted cDNA was pre-amplified using 2X Taqman PreAmp Master Mix (Applied Biosystems) and 50 nM of each primer pair in 5 μl reaction volume, according to the manufacturer’s directions. The cycling program was 10 min at 95°C followed by 16 cycles of 15 s at 95°C and 4 min at 60°C. At the end of this pre-amplification step, the reactions were diluted 1:5 (diluted pre-amplification samples). RT-qPCR on the dynamic array chips was conducted on the BioMark™ system (Fluidigm). Five μl sample pre-mix containing 2.5 μl of SsoFast EvaGreen Supermix with Low ROX (Bio-Rad), 0.25 μl of DNA Binding Dye Sample Loading Reagent (Fluidigm) and 2.25 μl of diluted pre-amplification samples (1:16 or 1:64 from the diluted pre-amplification samples from liver and backfat, respectively), as well as 5 μl assay mix containing 2.5 μl of Assay Loading Reagent (Fluidigm), 2.25 μl of DNA Suspension Buffer (Teknova) and 0.25 μl of 100 μM primer pairs (500 nM in the final reaction) were mixed inside the chip using the IFC controller MX (Fluidigm). The thermal cycle was 60s at 95°C followed by 30 cycles of 5 s at 96°C and 20s at 60°C. A dissociation curve was also drawn for each primer pair.

Data was collected using the Fluidigm Real-Time PCR analysis software 3.0.2 (Fluidigm) and analyzed using the DAG expression software 1.0.4.11 [91] applying the relative standard curve method (see Applied Biosystems user bulletin #2). Standard curves with a four-fold dilutions series (1/4, 1/16, 1/64, 1/256, 1/1024) of a pool of 10 cDNA samples were constructed for each gene to extrapolate the quantity values of the studied samples. The PCR efficiencies were almost 100% in both tissues for all the assays (Additional file 13: Table S10) with low coefficients of inter-assay variation of threshold cycle (<2.4% in liver and <3.5% in adipose tissue). Of the four endogenous genes tested (*ACTB, B2M, HPRT1, TBP*), *ACTB* and *TBP* were the genes with the most stable expression [89] in both tissues. The normalized quantity values of each sample and assay were used to compare our data.

All the primers used in this study were designed using PrimerExpress 2.0 software (Applied Biosystems) and are shown in Additional file 13: Table S10. Prior to perform the Fluidigm Real-Time PCR, all the assays were tested for PCR specificity in an ABI PRISM 7900 Sequence Detection System (Applied Biosystems) using two-fold dilutions (1/20, 1/200) of a pool of ten cDNA samples and a minus RT control to check the presence of DNA contamination. Melting curve analysis was performed for all the assays.

### Data availability

The relevant information and full data sets are included as additional files.

## Competing interests

The authors declare that they have no competing of interests.

## Authors’ contributions

YR-C, AR and JMF conceived and designed the experiment. JMF was the principal investigator of the project. YR-C, MB, MF, MP-E and AR performed the data analysis. AE annotated the SNPs. YR-C, MB, MF, AR and JM drafted the manuscript. AC, JN, AF, MP-E and JMF collected the samples. AC and MB performed DNA and RNA isolation. MB and AC performed the qPCR and RT-PCR assays. All authors read and approved the final manuscript.

## Supplementary Material

Additional file 1: Table S1 Positional concordance among the ten top connected nodes and QTL deposited in the pig QTL database for fatness related traits. Click here for file

Additional file 2: Table S2 Overrepresented GO terms indentified in the network using BinGO Cytoscape plugin. Click here for file

Additional file 3: Table S3 Overrepresented pathways identified with Fatigo. Click here for file

Additional file 4: Table S4 Complete list of the 74 available TF in the AWM. Click here for file

Additional file 5: Figure S1 Overrepresented pathways related to the 730 AWM-target genes according ClueGO results. ClueGO visualizes the terms in a functionally grouped annotation network, reflecting the relationships between the terms (based on the similarity of their associated genes). The size of the nodes reflects the statistical significance of the terms. The group leading term is the most significant term of the group. Click here for file

Additional file 6: Figure S2 Gene co-expression network in liver (A) and adipose (B) tissue. Nodes color relate to the functional classification of genes as follows: TF (red nodes), lipid metabolism (blue nodes), carbohydrate metabolism (green), development process (orange) and white nodes represent genes with others functional classification. Click here for file

Additional file 7: Table S5 Predicted AWM gene-gene interactions confirmed by the co-expression analysis in both liver and adipose tissues. Click here for file

Additional file 8: Table S6 List of the 39 AWM-predicted target genes that have been recently reported in two large-scale meta-analysis studies for plasma lipids in humans. Click here for file

Additional file 9: Table S7 List of the 30 genes involved in lipid metabolism predicted as target genes of *EP300.* Click here for file

Additional file 10: Figure S3 Protein-protein interaction among *EP300, FHL2* and *NCOA2* with *ESR1* and *AR* inferred from String database. Click here for file

Additional file 11: Table S8 Brief description, mean, standard deviation (SD) and estimated heritability (h^2^) of the 15 analyzed traits. Click here for file

Additional file 12: Table S9 List of 34 TF retained after Step 3 for choosing the threshold. Click here for file

Additional file 13: Table S10 Primers used in the experimental validation by real-time PCR. Click here for file
