# Supplementary material for: From SNP co-association to RNA co-expression: Novel insights into gene networks for intramuscular fatty acid composition in porcine
Source: BMC Genomics. 2014 Mar 26;15:232. doi: 10.1186/1471-2164-15-232 (PMC3987146; doi:10.1186/1471-2164-15-232)
Supplement: Additional file 1: Table S1 — Positional concordance among the ten top connected nodes and QTL deposited in the pig QTL database for fatness related traits. [file 1471-2164-15-232-S1.doc]

**Additional files**

**Additional file 1: Table S1**. Positional concordance among the ten top connected nodes and QTL deposited in the pig QTL database for fatness related traits.

| **SNP** | **Reference** | **Trait** | **QTL_Id** |
| --- | --- | --- | --- |
| ALGA0061664 | Milan, D. et al., 2002 | Backfat weight | 422 |
|  |  | Percentage of backfat and leaf fat in carcass | 423 |
|  | Edwards, DB. et al., 2008 | Backfat at last rib (10 weeks) | 3220 |
|  | Uddin, MJ. et al., 2011 | Triglyceride level | 17722 |
|  | Guo, YM. et al., 2008 | Backfat at mid-back | 5214 |
| H3GA0024739 | Duthie, C. et al., 2008 | Ham weight | 5129 |
|  |  | Lipid accretion rate | 5153 |
|  | Kim, JJ. et al., 2005 | Carcass weight (cold) | 2950 |
| DIAS0001129 | Uddin, MJ. et al., 2011 | LDL cholesterol | 17725 |
|  | Liu, G. et al., 2007 | Fat area | 5720 |
|  | Sanchez, MP. et al., 2007 | Monounsaturated fatty acid percentage | 7437 |
|  |  | Oleic acid percentage | 7435 |
|  | Rückert C. & Bennewitz J. 2010 | Backfat at last rib | 16840 |
| ASGA0021047 | Kim, Y. et al., 2006 | Linoleic acid percentage | 4203 |
|  | Perez-Enciso, M. et al., 2000 | Linoleic acid percentage | 535 |
|  | Uemoto, Y. et al., 2012 | Oleic acid percentage | 15875 |
|  | Grindflek, E. et al., 2001 | Margaric acid percentage | 158 |
|  | Paszek, AA. et al., 2001 | Intramuscular fat content | 3871 |
|  | Nagamine, Y. et al., 2003 | Backfat thickness - ultrasound | 3759 |
| MARC0045253 | Lee, C. et al., 2003 | Linoleic acid percentage | 686 |
|  | Malek, M. et al., 2001 | Muscle fat content | 79 |
|  | Grapes L. & Rothschild MF. 2006 | Marbling | 4020 |
|  | Rückert, C. et al., 2012 | Adipocyte diameter | 10028 |
| ALGA0025325 | de Koning, DJ et al., 2009 | Intramuscular fat content | 313 |
|  | Uddin, MJ. et al., 2011 | HDL/LDL ratio | 17717 |
|  | Knott, SA. et al., 2002 | Fat percentage in carcass | 459 |
|  |  | Abdominal fat weight | 261 |
|  | Geldermann, H. et al., 2010 | Adipocyte diameter | 12827 |
|  | Bidanel, JP. et al., 2001 | Backfat (40 and 60 kg live weight) | 200, 201 |
|  | Wimmers, K. et al., 2002 | Backfat at mid-back | 463 |
| ALGA0117149 | Nii, M. et al., 2006 | Palmitoleic acid percentage | 3109 |
|  |  | Stearic acid percentage | 3111 |
|  | Liu, G. et al., 2007 | Average backfat thickness | 5670 |
| ALGA0059185 | Guo, YM. et al., 2008 | Backfat at mid-back | 5247 |
|  | Uemoto, Y. et al., 2012 | Linoleic acid percentage | 15861 |
|  | Rohrer GA. & Keele JW. 1998 | Backfat at first rib | 21 |
|  | Jong-Joo Kim. et al., 2005 | Backfat at last rib | 2952 |
| ASGA0008154 | Milan, D. et al., 2002 | Backfat weight | 401 |
|  | Guo, YM. et al., 2008 | Backfat at mid-back | 5241 |
|  | Bidanel, JP. et al., 2001 | Backfat (13, 17 weeks of age) | 192, 193 |
|  | Bidanel, JP. et al., 2001 | Backfat (40 kg , 60 kg live weight) | 195, 196 |
| DIAS0004697 | H.J. van Wijk. et al., 2006 | Marbling | 3079 |
|  | Rückert C. & Bennewitz J. 2010 | Backfat at last rib | 16841 |
|  | Uemoto, Y. et a.,l 2012 | Stearic acid percentage | 15870 |
|  |  | Linoleic acid percentage | 15871 |
|  | Liu, Guisheng. et al., 2008 | Backfat above muscle dorsi | 5991 |

**Reference:**

- Milan D, Bidanel JP, Iannuccelli N, Riquet J, Amigues Y, et al. (2002) Detection of quantitative trait loci for carcass composition traits in pigs. Genet Sel Evol 34: 705-728.

- Edwards DB, Ernst CW, Tempelman RJ, Rosa GJ, Raney NE, et al. (2008) Quantitative trait loci mapping in an F2 Duroc x Pietrain resource population: I. Growth traits. J Anim Sci 86: 241-253.

- Uddin MJ, Duy do N, Cinar MU, Tesfaye D, Tholen E, et al. (2011) Detection of quantitative trait loci affecting serum cholesterol, LDL, HDL, and triglyceride in pigs. BMC Genet 12: 62.

- Rohrer GA, Keele JW (1998) Identification of quantitative trait loci affecting carcass composition in swine: I. Fat deposition traits. J Anim Sci 76: 2247-2254.

- Guo YM, Lee GJ, Archibald AL, Haley CS (2008) Quantitative trait loci for production traits in pigs: a combined analysis of two Meishan x Large White populations. Anim Genet 39: 486-495.

- Duthie C, Simm G, Doeschl-Wilson A, Kalm E, Knap PW, et al. (2008) Quantitative trait loci for chemical body composition traits in pigs and their positional associations with body tissues, growth and feed intake. Animal Genetics 39: 130-140.

- Kim J-J, Zhao H., THOMSEN H, ROTHSCHILD MF, DEKKERS JCM (2005) Combined line-cross and half-sib QTL analysis of crosses between outbred lines. Genetics Research 85: 235-248.

- Liu G, Jennen DGJ, Tholen E, Juengst H, Kleinwächter T, et al. (2007) A genome scan reveals QTL for growth, fatness, leanness and meat quality in a Duroc-Pietrain resource population. Animal Genetics 38: 241-252.

- Sanchez MP, Iannuccelli N, Basso B, Bidanel JP, Billon Y, et al. (2007) Identification of QTL with effects on intramuscular fat content and fatty acid composition in a Duroc x Large White cross. BMC Genet 8: 55.

- Ruckert C, Bennewitz J (2010) Joint QTL analysis of three connected F2-crosses in pigs. Genet Sel Evol 42: 40.

- Nagamine Y, Haley CS, Sewalem A, Visscher PM (2003) Quantitative Trait Loci Variation for Growth and Obesity Between and Within Lines of Pigs (Sus scrofa). Genetics 164: 629-635.

- Paszek AA, Wilkie PJ, Flickinger GH, Miller LM, Louis CF, et al. (2001) Interval mapping of carcass and meat quality traits in a divergent swine cross. Anim Biotechnol 12: 155-165.

- Kim Y, Kong M, Nam YJ, Lee C (2006) A quantitative trait locus for oleic fatty acid content on Sus scrofa chromosome 7. J Hered 97: 535-537.

- Perez-Enciso M, Clop A, Noguera JL, Ovilo C, Coll A, et al. (2000) A QTL on pig chromosome 4 affects fatty acid metabolism: evidence from an Iberian by Landrace intercross. J Anim Sci 78: 2525-2531.

- Grindflek E, Szyda J, Liu Z, Lien S (2001) Detection of quantitative trait loci for meat quality in a commercial slaughter pig cross. Mamm Genome 12: 299-304.

- Uemoto Y, Soma Y, Sato S, Ishida M, Shibata T, et al. (2012) Genome-wide mapping for fatty acid composition and melting point of fat in a purebred Duroc pig population. Anim Genet 43: 27-34.

- Lee C, Chung Y, Kim JH (2003) Quantitative trait loci mapping for fatty acid contents in the backfat on porcine chromosomes 1, 13, and 18. Mol Cells 15: 62-67.

- Malek M, Dekkers JC, Lee HK, Baas TJ, Prusa K, et al. (2001) A molecular genome scan analysis to identify chromosomal regions influencing economic traits in the pig. II. Meat and muscle composition. Mamm Genome 12: 637-645.

- Grapes L, Rothschild MF (2006) Investigation of a QTL region for loin eye area and fatness on pig chromosome 1. Mamm Genome 17: 657-668.

- Ruckert C, Stratz P, Preuss S, Bennewitz J (2012) Mapping quantitative trait loci for metabolic and cytological fatness traits of connected F2 crosses in pigs. J Anim Sci 90: 399-409.

- de Koning DJ, Janss LLG, Rattink AP, van Oers PAM, de Vries BJ, et al. (1999) Detection of Quantitative Trait Loci for Backfat Thickness and Intramuscular Fat Content in Pigs (Sus scrofa). Genetics 152: 1679-1690.

- Knott SA, Nyström PE, Andersson-Eklund L, Stern S, Marklund L, et al. (2002) Approaches to interval mapping of QTL in a multigeneration pedigree: the example of porcine chromosome 4. Animal Genetics 33: 26-32.

- Geldermann H, Cepica S, Stratil A, Bartenschlager H, Preuss S (2010) Genome-wide mapping of quantitative trait loci for fatness, fat cell characteristics and fat metabolism in three porcine F2 crosses. Genet Sel Evol 42: 31.

- Bidanel JP, Milan D, Iannuccelli N, Amigues Y, Boscher MY, et al. (2001) Detection of quantitative trait loci for growth and fatness in pigs. Genet Sel Evol 33: 289-309.

- Wimmers K, Murani E, Ponsuksili S, Yerle M, Schellander K (2002) Detection of quantitative trait loci for carcass traits in the pig by using AFLP. Mamm Genome 13: 206-210.

- Nii M, Hayashi T, Tani F, Niki A, Mori N, et al. (2006) Quantitative trait loci mapping for fatty acid composition traits in perirenal and back fat using a Japanese wild boar _ Large White intercross. Animal Genetics 37: 342-347.

- Liu G, Kim JJ, Jonas E, Wimmers K, Ponsuksili S, et al. (2008) Combined line-cross and half-sib QTL analysis in Duroc-Pietrain population. Mamm Genome 19: 429-438.

- de Koning DJ, Rattink AP, Harlizius B, Groenen MAM, Brascamp EW, et al. (2001) Detection and characterization of quantitative trait loci for growth and reproduction traits in pigs. Livestock Production Science 72: 185-198.

- Ruckert C, Stratz P, Preuss S, Bennewitz J (2012) Mapping quantitative trait loci for metabolic and cytological fatness traits of connected F2 crosses in pigs. Journal of Animal Science 90: 399-409.

- van Wijk HJ, Dibbits B, Baron EE, Brings AD, Harlizius B, et al. (2006) Identification of quantitative trait loci for carcass composition and pork quality traits in a commercial finishing cross. Journal of Animal Science 84: 789-799.
