# Supplementary material for: From SNP co-association to RNA co-expression: Novel insights into gene networks for intramuscular fatty acid composition in porcine
Source: BMC Genomics. 2014 Mar 26;15:232. doi: 10.1186/1471-2164-15-232 (PMC3987146; doi:10.1186/1471-2164-15-232)
Supplement: Additional file 2: Table S2 — Overrepresented GO terms indentified in the network using BinGO Cytoscape plugin. [file 1471-2164-15-232-S2.doc]

**Additional file 2: Table S2**. Overrepresented GO terms indentified in the network.

| **GO term** | **Description** | **Nr. Genes** | **P-value** | **FDR** |
| --- | --- | --- | --- | --- |
| GO:0016043 | cellular component organization | 294 | 4.02E-006 | 3.95E-002 |
| GO:0071840 | cellular component organization or biogenesis | 294 | 7.34E-006 | 3.60E-002 |
| GO:0030030 | cell projection organization | 64 | 8.07E-006 | 2.64E-002 |
| GO:0045664 | regulation of neuron differentiation | 50 | 1.09E-005 | 2.68E-002 |
| GO:0048812 | neuron projection morphogenesis | 29 | 1.31E-005 | 2.58E-002 |
| GO:0010975 | regulation of neuron projection development | 39 | 4.25E-005 | 5.97E-002 |
| GO:0021952 | central nervous system projection neuron axonogenesis | 9 | 4.88E-005 | 5.99E-002 |
| GO:0007409 | axonogenesis | 20 | 6.67E-005 | 7.27E-002 |
| GO:0048858 | cell projection morphogenesis | 30 | 9.59E-005 | 9.42E-002 |
| GO:0050767 | regulation of neurogenesis | 55 | 1.11E-004 | 9.88E-002 |
| GO:0006631 | fatty acid metabolic process | 85 | 5.89E-004 | 1.03E-002 |
| GO:0006665 | sphingolipid metabolic process | 27 | 7.45E-004 | 1.16E-002 |
| GO:0046486 | glycerolipid metabolic process | 30 | 1.24E-003 | 1.66E-002 |
| GO:0006636 | unsaturated fatty acid biosynthetic process | 14 | 2.13E-003 | 2.27E-002 |
| GO:0050852 | T cell receptor signaling pathway | 48 | 5.94E-003 | 4.59E-002 |
