# Supplementary material for: From SNP co-association to RNA co-expression: Novel insights into gene networks for intramuscular fatty acid composition in porcine
Source: BMC Genomics. 2014 Mar 26;15:232. doi: 10.1186/1471-2164-15-232 (PMC3987146; doi:10.1186/1471-2164-15-232)
Supplement: Additional file 3: Table S3 — Overrepresented pathways identified with Fatigo. [file 1471-2164-15-232-S3.doc]

**Additional file 3: Table S3**. Overrepresented pathways identified with Fatigo.

| **Name** | **Term** | **Genes x Pathway** | **P-value** | **FDR** |
| --- | --- | --- | --- | --- |
| Pathways in cancer | hsa05200 | 29 | 7.33E-04 | 2.06E-02 |
| Regulation of actin cytoskeleton | hsa04810 | 20 | 1.64E-03 | 2.69E-02 |
| Focal adhesion | hsa04510 | 20 | 1.54E-03 | 2.69E-02 |
| Chemokine signaling pathway | hsa04062 | 18 | 2.84E-03 | 4.24E-02 |
| Tight junction | hsa04530 | 15 | 1.66E-03 | 2.69E-02 |
| Axon guidance | hsa04360 | 16 | 2.25E-04 | 1.09E-02 |
| Neurotrophin signaling pathway | hsa04722 | 15 | 7.00E-04 | 2.06E-02 |
| Leukocyte transendothelial migration | hsa04670 | 17 | 1.81E-05 | 2.35E-03 |
| Colorectal cancer | hsa05210 | 11 | 1.56E-03 | 2.69E-02 |
| Phosphatidylinositol signaling system | hsa04070 | 11 | 7.43E-04 | 2.06E-02 |
| Arrhythmogenic right ventricular cardiomyopathy | hsa05412 | 13 | 2.42E-05 | 2.35E-03 |
| Renal cell carcinoma | hsa05211 | 10 | 1.26E-03 | 2.69E-02 |
| Acute myeloid leukemia | hsa05221 | 10 | 1.95E-04 | 1.09E-02 |
| Inositol phosphate metabolism | hsa00562 | 8 | 3.29E-03 | 4.56E-02 |
