# Supplementary material for: From SNP co-association to RNA co-expression: Novel insights into gene networks for intramuscular fatty acid composition in porcine
Source: BMC Genomics. 2014 Mar 26;15:232. doi: 10.1186/1471-2164-15-232 (PMC3987146; doi:10.1186/1471-2164-15-232)
Supplement: Additional file 4: Table S4 — Complete list of the 74 available TF in the AWM. [file 1471-2164-15-232-S4.doc]

**Additional file 4: Table S4**. Complete list of the 74 available TF in the AWM.

| **Symbol** | **Description** |
| --- | --- |
| ACTR5 | ARP5 actin-related protein 5 homolog (yeast) |
| AFF2 | AF4/FMR2 family, member 2 |
| AFF3 | AF4/FMR2 family, member 3 |
| ARID1B | AT rich interactive domain 1B (SWI1-like) |
| ARNT | aryl hydrocarbon receptor nuclear translocator |
| ATF6 | activating transcription factor 6 |
| BCL9 | B-cell CLL/lymphoma 9 |
| BDP1 | B double prime 1, subunit of RNA polymerase III transcription initiation factor IIIB |
| BRPF1 | bromodomain and PHD finger containing, 1 |
| CAR | nuclear receptor subfamily 1, group I, member 3 |
| CCRN4L | CCR4 carbon catabolite repression 4-like (S. cerevisiae) |
| CHD1 | chromodomain helicase DNA binding protein 1 |
| CIITA | class II, major histocompatibility complex, transactivator |
| COPS5 | COP9 constitutive photomorphogenic homolog subunit 5 (Arabidopsis) |
| CREB5 | cAMP responsive element binding protein 5 |
| DDX20 | DEAD (Asp-Glu-Ala-Asp) box polypeptide 20 |
| ELL2 | elongation factor, RNA polymerase II, 2 |
| EP300 | E1A binding protein p300 |
| ETV6 | ets variant 6 |
| FHL2 | four and a half LIM domains 2 |
| GFI1B | growth factor independent 1B transcription repressor |
| GTF2A1 | general transcription factor IIA, 1, 19/37kDa |
| HIVEP1 | human immunodeficiency virus type I enhancer binding protein 1 |
| HMBOX1 | homeobox containing 1 |
| HMGB1 | high mobility group box 1 |
| HNF1A | HNF1 homeobox A |
| JARID2 | jumonji, AT rich interactive domain 2 |
| MAML3 | mastermind-like 3 (Drosophila) |
| MAX | MYC associated factor X |
| MBD2 | methyl-CpG binding domain protein 2 |
| MDFIC | MyoD family inhibitor domain containing |
| MED12L | mediator complex subunit 12-like |
| MTF2 | metal response element binding transcription factor 2 |
| MYB | v-myb myeloblastosis viral oncogene homolog (avian) |
| MYC | v-myc myelocytomatosis viral oncogene homolog (avian) |
| MYST4 | K(lysine) acetyltransferase 6B |
| NCOA2 | nuclear receptor coactivator 2 |
| NR2C2 | nuclear receptor subfamily 2, group C, member 2 |
| NR2E1 | nuclear receptor subfamily 2, group E, member 1 |
| NR2F6 | nuclear receptor subfamily 2, group F, member 6 |
| PBX1 | pre-B-cell leukemia homeobox 1 |
| PHF17 | PHD finger protein 17 |
| PHTF1 | putative homeodomain transcription factor 1 |
| PMF1 | polyamine-modulated factor 1 |
| PPARG | peroxisome proliferator-activated receptor gamma |
| PPARGC1B | peroxisome proliferator-activated receptor gamma, coactivator 1 beta |
| PROX1 | prospero homeobox 1 |
| RORC | RAR-related orphan receptor C |
| RUNX1T1 | runt-related transcription factor 1; translocated to, 1 (cyclin D-related) |
| RUVBL2 | RuvB-like 2 (E. coli) |
| SALL1 | sal-like 1 (Drosophila) |
| SETD7 | SET domain containing (lysine methyltransferase) 7 |
| SLC30A9 | solute carrier family 30 (zinc transporter), member 9 |
| SMARCA5 | SWI/SNF related, matrix associated, actin dependent regulator of chromatin, subfamily a, member 5 |
| SMARCAD1 | SWI/SNF-related, matrix-associated actin-dependent regulator of chromatin, subfamily a, containing DEAD/H box 1 |
| ST18 | suppression of tumorigenicity 18 (breast carcinoma) (zinc finger protein) |
| STAT2 | signal transducer and activator of transcription 2, 113kDa |
| STAT4 | signal transducer and activator of transcription 4 |
| T | T, brachyury homolog (mouse) |
| TAF2 | TAF2 RNA polymerase II, TATA box binding protein (TBP)-associated factor, 150kDa |
| TBX18 | T-box 18 |
| TCEA1 | transcription elongation factor A (SII), 1 |
| TCF7L1 | transcription factor 7-like 1 (T-cell specific, HMG-box) |
| TCF7L2 | transcription factor 7-like 2 (T-cell specific, HMG-box) |
| TRRAP | transformation/transcription domain-associated protein |
| TSC22D1 | TSC22 domain family, member 1 |
| VPS72 | vacuolar protein sorting 72 homolog (S. cerevisiae) |
| WWTR1 | WW domain containing transcription regulator 1 |
| ZFHX4 | zinc finger homeobox 4 |
| ZFPM2 | zinc finger protein, multitype 2 |
| ZNF213 | zinc finger protein 213 |
| ZNF395 | zinc finger protein 395 |
| ZNF423 | zinc finger protein 423 |
| ZNF473 | zinc finger protein 473 |
