# Supplementary material for: From SNP co-association to RNA co-expression: Novel insights into gene networks for intramuscular fatty acid composition in porcine
Source: BMC Genomics. 2014 Mar 26;15:232. doi: 10.1186/1471-2164-15-232 (PMC3987146; doi:10.1186/1471-2164-15-232)
Supplement: Additional file 5: Figure S1 — Overrepresented pathways related to the 730 AWM-target genes according ClueGO results. ClueGO visualizes the terms in a functionally grouped annotation network, reflecting the relationships between the terms (based on the similarity of their associated genes). The size of the nodes reflects the statistical significance of the terms. The group leading term is the most significant term of the group. [file 1471-2164-15-232-S5.doc]

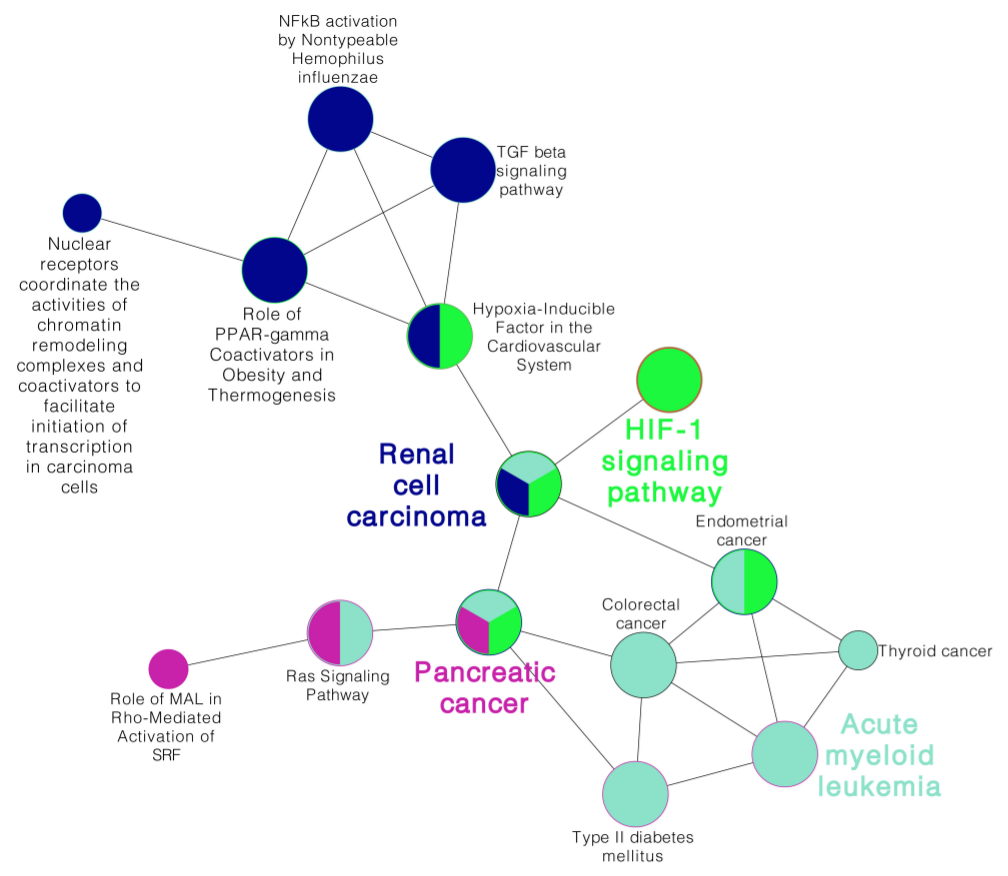


**Additional file 5: Figure S1.** Overrepresented pathways related to the 730 AWM-target genes according ClueGO results. ClueGO visualizes the terms in a functionally grouped annotation network, reflecting the relationships between the terms (based on the similarity of their associated genes). The size of the nodes reflects the statistical significance of the terms. The group leading term is the most significant term of the group.
