# Supplementary material for: From SNP co-association to RNA co-expression: Novel insights into gene networks for intramuscular fatty acid composition in porcine
Source: BMC Genomics. 2014 Mar 26;15:232. doi: 10.1186/1471-2164-15-232 (PMC3987146; doi:10.1186/1471-2164-15-232)
Supplement: Additional file 6: Figure S2 — Gene co-expression network in liver (A) and adipose (B) tissue. Nodes color relate to the functional classification of genes as follows: TF (red nodes), lipid metabolism (blue nodes), carbohydrate metabolism (green), development process (orange) and white nodes represent genes with others functional classification. [file 1471-2164-15-232-S6.doc]

**
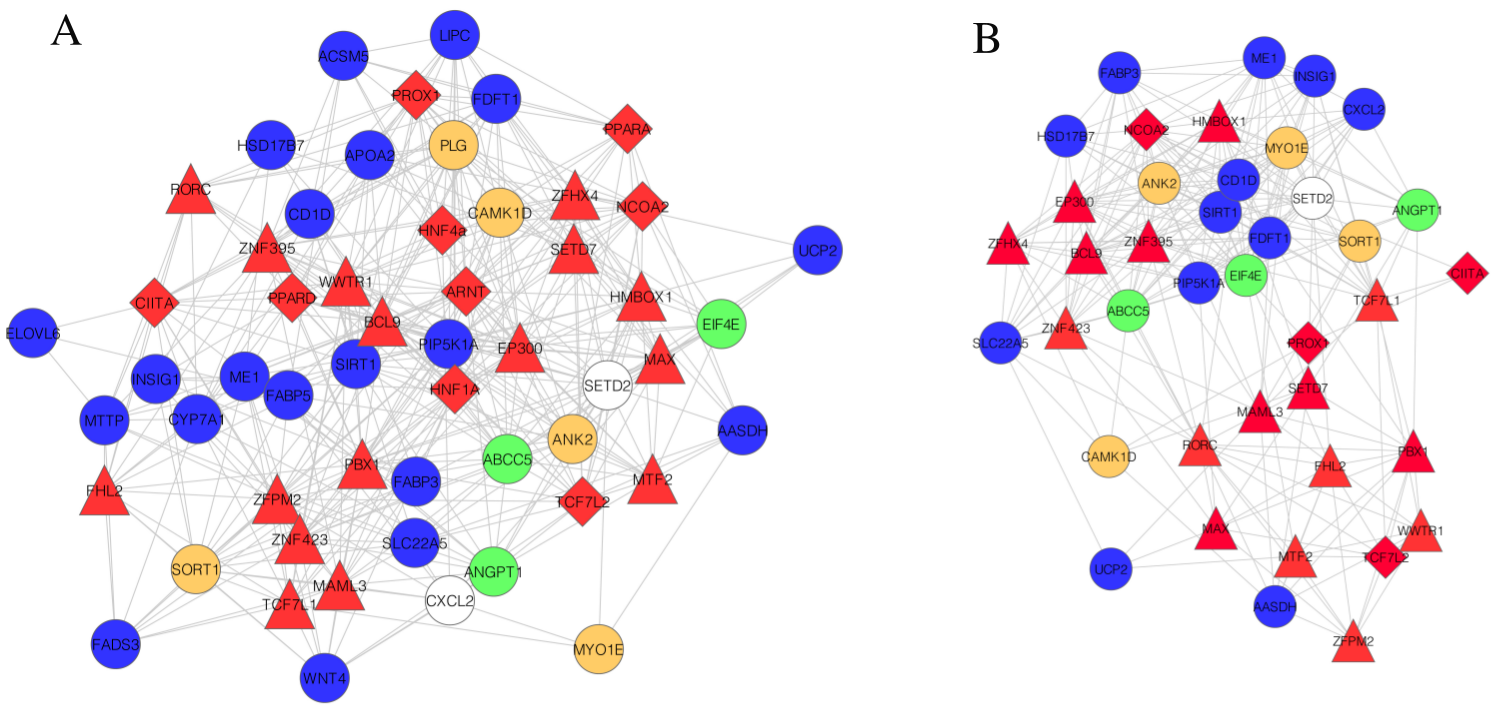
**

**Additional file 6: Figure S2.** Gene co-expression network in liver (A) and adipose (B) tissue. Nodes color relate to the functional classification of genes as follows: TF (red nodes), lipid metabolism (blue nodes), carbohydrate metabolism (green), development process (orange) and white nodes represent genes with others functional classification.
