# Supplementary material for: From SNP co-association to RNA co-expression: Novel insights into gene networks for intramuscular fatty acid composition in porcine
Source: BMC Genomics. 2014 Mar 26;15:232. doi: 10.1186/1471-2164-15-232 (PMC3987146; doi:10.1186/1471-2164-15-232)
Supplement: Additional file 7: Table S5 — Predicted AWM gene-gene interactions confirmed by the co-expression analysis in both liver and adipose tissues. [file 1471-2164-15-232-S7.doc]

**Additional file 7: Table S5**. Predicted AWM gene-gene interactions confirmed by the co-expression analysis in both liver and adipose tissues.

| **From** | **To** |
| --- | --- |
| ZFPM2 | PBX1 |
| ABCC5 | SETD2 |
| ANK2 | EP300 |
| HMBOX1 | ABCC5 |
| UCP2 | MAX |
| PBX1 | TCF7L2 |
| PBX1 | SORT1 |
| INSIG1 | SORT1 |
| RORC | BCL9 |
| ZFHX4 | NCOA2 |
| BCL9 | ZNF423 |
| SETD7 | MAX |
| HMBOX1 | FDFT1 |
| ANGPT1 | CXCL2 |
| HMBOX1 | NCOA2 |
| FABP3 | ZNF423 |
| BCL9 | CD1D |
| ZNF395 | ZFHX4 |
| FDFT1 | ZFHX4 |
| NCOA2 | SETD2 |
| RORC | FHL2 |
| ANGPT1 | PBX1 |
| MTF2 | AASDH |
| ZFPM2 | TCF7L2 |
| HMBOX1 | SETD2 |
| PIP5K1A | BCL9 |
| FABP3 | INSIG1 |
| EIF4E | EP300 |
| SLC22A5 | ABCC5 |
| EP300 | SETD2 |
