# Supplementary material for: From SNP co-association to RNA co-expression: Novel insights into gene networks for intramuscular fatty acid composition in porcine
Source: BMC Genomics. 2014 Mar 26;15:232. doi: 10.1186/1471-2164-15-232 (PMC3987146; doi:10.1186/1471-2164-15-232)
Supplement: Additional file 8: Table S6 — List of the 39 AWM-predicted target genes that have been recently reported in two large-scale meta-analysis studies for plasma lipids in humans. [file 1471-2164-15-232-S8.doc]

**Additional file 8: Table S6**. List of the 39 AWM-predicted target genes that have been recently reported in two large-scale meta-analysis studies for plasma lipids in humans.

| **GeneId** | **AWM-predicted regulator** | **Description** |
| --- | --- | --- |
| ABCC2 | NCOA2, FHL2 | ATP-binding cassette, sub-family C (CFTR/MRP), member 2 |
| ABCC5 | NCOA2 | ATP-binding cassette, sub-family C (CFTR/MRP), member 5 |
| AFF2 | FHL2 | AF4/FMR2 family, member 2 |
| APP | NCOA2 | amyloid beta (A4) precursor protein |
| ARNT | NCOA2, FHL2 | aryl hydrocarbon receptor nuclear translocator |
| ARRB1 | EP300 | arrestin, beta 1 |
| CCRN4L | NCOA2 | CCR4 carbon catabolite repression 4-like (S. cerevisiae) |
| FAF1 | FHL2 | Fas (TNFRSF6) associated factor 1 |
| FDFT1 | NCOA2 | farnesyl-diphosphate farnesyltransferase 1 |
| FLT1 | FHL2 | fms-related tyrosine kinase 1 (vascular endothelial growth factor/vascular permeability factor receptor) |
| GAS7 | FHL2 | growth arrest-specific 7 |
| IL6R | NCOA2, FHL2 | interleukin 6 receptor |
| LIPC | FHL2 | lipase, hepatic |
| MMP16 | NCOA2 | matrix metallopeptidase 16 (membrane-inserted) |
| NOS1AP | FHL2 | nitric oxide synthase 1 (neuronal) adaptor protein |
| TGFBR3 | NCOA2 | transforming growth factor, beta receptor III |
| ABCB11 | FHL2 | ATP-binding cassette, sub-family B (MDR/TAP), member 11 |
| ADCY2 | NCOA2, EP300 | adenylate cyclase 2 (brain) |
| COL9A1 | EP300 | collagen, type IX, alpha 1 |
| ECHS1 | EP300 | enoyl CoA hydratase, short chain, 1, mitochondrial |
| HTR2A | FHL2 | 5-hydroxytryptamine (serotonin) receptor 2A, G protein-coupled |
| IKBKB | NCOA2, FHL2 | inhibitor of kappa light polypeptide gene enhancer in B-cells, kinase beta |
| IL12A | EP300 | interleukin 12A (natural killer cell stimulatory factor 1, cytotoxic lymphocyte maturation factor 1, p35) |
| RYR2 | NCOA2 | ryanodine receptor 2 (cardiac) |
| SORT1 | NCOA2 | sortilin 1 |
| TCF7L2 | NCOA2 | transcription factor 7-like 2 (T-cell specific, HMG-box) |
| TPM1 | EP300 | tropomyosin 1 (alpha) |
| FABP3 | NCOA2 | fatty acid binding protein 3, muscle and heart (mammary-derived growth inhibitor) |
| TTR | FHL2 | transthyretin |
| ADORA3 | NCOA2 | adenosine A3 receptor |
| MCL1 | FHL2 | myeloid cell leukemia sequence 1 (BCL2-related) |
| NTRK2 | FHL2 | neurotrophic tyrosine kinase, receptor, type 2 |
| VCAM1 | NCOA2 | vascular cell adhesion molecule 1 |
| WNK1 | FHL2 | WNK lysine deficient protein kinase 1 |
| MMP9 | EP300 | matrix metallopeptidase 9 (gelatinase B, 92kDa gelatinase, 92kDa type IV collagenase) |
| XPO7 | NCOA2 | exportin 7 |
| HNF1A | NCOA2 | HNF1 homeobox A |
| EVI5 | NCOA2 | ecotropic viral integration site 5 |
| FRK | NCOA2 | fyn-related kinase |
