# Supplementary material for: From SNP co-association to RNA co-expression: Novel insights into gene networks for intramuscular fatty acid composition in porcine
Source: BMC Genomics. 2014 Mar 26;15:232. doi: 10.1186/1471-2164-15-232 (PMC3987146; doi:10.1186/1471-2164-15-232)
Supplement: Additional file 9: Table S7 — List of the 30 genes involved in lipid metabolism predicted as target genes of EP300. [file 1471-2164-15-232-S9.doc]

**Additional file 9: Table S7**. List of the 30 genes involved in lipid metabolism predicted as target genes of *EP300*

| **GeneId** | **Description** |
| --- | --- |
| ELOVL6 | ELOVL fatty acid elongase 6 |
| ACSM5 | acyl-CoA synthetase medium-chain family member 5 |
| MTTP | microsomal triglyceride transfer protein |
| MMP9 | matrix metallopeptidase 9 (gelatinase B, 92kDa gelatinase, 92kDa type IV collagenase) |
| CYP2E1 | cytochrome P450, family 2, subfamily E, polypeptide 1 |
| UCP2 | uncoupling protein 2 (mitochondrial, proton carrier) |
| SLC22A5 | solute carrier family 22 (organic cation/carnitine transporter), member 5 |
| MYO5A | myosin VA (heavy chain 12, myoxin) |
| NR2E1 | nuclear receptor subfamily 2, group E, member 1 |
| LEP | leptin |
| SGPP1 | sphingosine-1-phosphate phosphatase 1 |
| PLCD1 | phospholipase C, delta 1 |
| ADCY2 | adenylate cyclase 2 (brain) |
| GL | protein phosphatase 1, regulatory subunit 3B |
| ACSM2B | acyl-CoA synthetase medium-chain family member 2B |
| NOX5 | NADPH oxidase, EF-hand calcium binding domain 5 |
| MYD88 | myeloid differentiation primary response gene (88) |
| CPS1 | carbamoyl-phosphate synthase 1, mitochondrial |
| ST8SIA3 | ST8 alpha-N-acetyl-neuraminide alpha-2,8-sialyltransferase 3 |
| PITPNC1 | phosphatidylinositol transfer protein, cytoplasmic 1 |
| LGALS8 | lectin, galactoside-binding, soluble, 8 |
| NDST3 | N-deacetylase/N-sulfotransferase (heparan glucosaminyl) 3 |
| HSD3B7 | hydroxy-delta-5-steroid dehydrogenase, 3 beta- and steroid delta-isomerase 7 |
| DDHD1 | DDHD domain containing 1 |
| ALDH1L2 | aldehyde dehydrogenase 1 family, member L2 |
| LPCAT2 | lysophosphatidylcholine acyltransferase 2 |
| ECHS1 | enoyl CoA hydratase, short chain, 1, mitochondrial |
| CWH43 | cell wall biogenesis 43 C-terminal homolog (S. cerevisiae) |
| ARNT | aryl hydrocarbon receptor nuclear translocator |
| SOS2 | son of sevenless homolog 2 (Drosophila) |
