# Supplementary material for: From SNP co-association to RNA co-expression: Novel insights into gene networks for intramuscular fatty acid composition in porcine
Source: BMC Genomics. 2014 Mar 26;15:232. doi: 10.1186/1471-2164-15-232 (PMC3987146; doi:10.1186/1471-2164-15-232)
Supplement: Additional file 10: Figure S3 — Protein-protein interaction among EP300, FHL2 and NCOA2 with ESR1 and AR inferred from String database. [file 1471-2164-15-232-S10.doc]

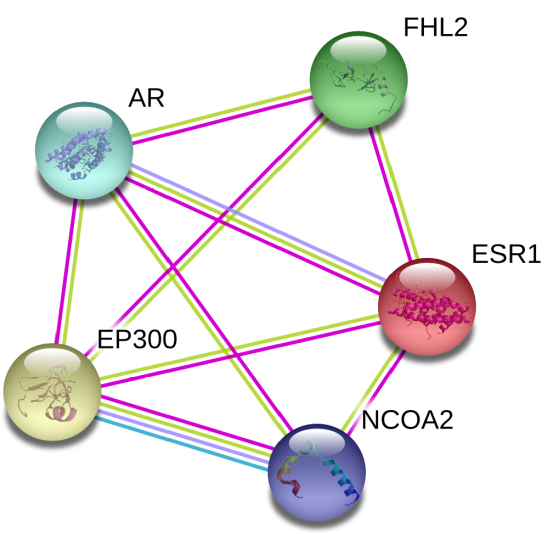

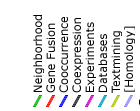


**Additional File 10: Figure S3**. Protein-protein interaction among *EP300, FHL2* and *NCOA2* with *ESR1* and *AR* inferred from String database
