# Supplementary material for: From SNP co-association to RNA co-expression: Novel insights into gene networks for intramuscular fatty acid composition in porcine
Source: BMC Genomics. 2014 Mar 26;15:232. doi: 10.1186/1471-2164-15-232 (PMC3987146; doi:10.1186/1471-2164-15-232)
Supplement: Additional file 11: Table S8 — Brief description, mean, standard deviation (SD) and estimated heritability (h2) of the 15 analyzed traits. [file 1471-2164-15-232-S11.doc]

**Additional File 11: Table S8.** Brief description, mean, standard deviation (SD) and estimated heritability (h^2^) of the 15 analyzed traits.

|  | **Trait** | **Label** | **Mean** | **SD** | **h^2^** |
| --- | --- | --- | --- | --- | --- |
| Saturated FA | C16:0 | Palmitic acid | 22.60 | 1.20 | 0.57 |
|  | C18:0 | Stearic acid | 14.18 | 1.03 | 0.24 |
| Monounsaturated FA | C16:1(n-7) | Palmitoleic acid | 2.50 | 0.39 | 0.55 |
|  | C18:1(n-9) | Oleic acid | 40.08 | 2.76 | 0.40 |
| Polyunsaturated FA | C18:2(n-6) | Linoleic acid | 10.35 | 2.37 | 0.22 |
|  | C18:3(n-3) | [α-Linolenic acid](http://en.wikipedia.org/wiki/Alpha-linolenic_acid) | 0.65 | 0.29 | 0.18 |
|  | C20:2(n-6) | [Eicosadienoic acid](http://en.wikipedia.org/w/index.php?title=Eicosadienoic_acid&action=edit&redlink=1) | 0.54 | 0.12 | 0.26 |
|  | C20:3(n-6) | Eicosatrienoic acid | 0.28 | 0.13 | 0.16 |
|  | C20:4(n-6) | Arachidonic acid | 1.54 | 0.73 | 0.40 |
| Metabolic ratios | ∑ SFA | Saturated FA | 38.47 | 1.63 | 0.53 |
|  | ∑ MUFA | Monounsaturated FA | 47.96 | 3.06 | 0.19 |
|  | ∑ PUFA | Polyunsaturated FA | 13.36 | 3.29 | 0.18 |
|  | UI | Unsaturated indices | 2.04 | 0.27 | 0.19 |
| FA ratio | C20:2(n-6)/C18:2(n-6) | Elongase activity | 0.05 | 0.01 | 0.39 |
| IMF | IMF | Percentage intramuscular fat | 2.03 | 0.63 | 0.42 |
