# Supplementary material for: From SNP co-association to RNA co-expression: Novel insights into gene networks for intramuscular fatty acid composition in porcine
Source: BMC Genomics. 2014 Mar 26;15:232. doi: 10.1186/1471-2164-15-232 (PMC3987146; doi:10.1186/1471-2164-15-232)
Supplement: Additional file 12: Table S9 — List of 34 TF retained after Step 3 for choosing the threshold. [file 1471-2164-15-232-S12.doc]

**Additional File 12: Table S9.** List of 34 TF retained after Step 3 for choosing the threshold (see Material and Methods section).

| **TF_Symbol** | **Description** |
| --- | --- |
| HNF1A | HNF1 homeobox A |
| PPARG | peroxisome proliferator-activated receptor gamma |
| PPARGC1B | peroxisome proliferator-activated receptor gamma, coactivator 1 beta |
| NR2C2 | nuclear receptor subfamily 2, group C, member 2 |
| NR2E1 | nuclear receptor subfamily 2, group E, member 1 |
| PBX1 | pre-B-cell leukemia homeobox 1 |
| PROX1 | prospero homeobox 1 |
| ARNT | aryl hydrocarbon receptor nuclear translocator |
| CREB5 | cAMP responsive element binding protein 5 |
| LASS3 | ceramide synthase 3 |
| RXRG | retinoid X receptor, gamma |
| NR0B1 | nuclear receptor subfamily 0, group B, member 1 |
| VDR | vitamin D (1,25- dihydroxyvitamin D3) receptor |
| TRERF1 | transcriptional regulating factor 1 |
| NCOA2 | nuclear receptor coactivator 2 |
| USF1 | upstream transcription factor 1 |
| MYC | v-myc myelocytomatosis viral oncogene homolog (avian) |
| ATF6 | activating transcription factor 6 |
| EPAS1 | endothelial PAS domain protein 1 |
| ESR1 | estrogen receptor 1 |
| NCOA1 | nuclear receptor coactivator 1 |
| NR1H3 | nuclear receptor subfamily 1, group H, member 3 |
| NR1H4 | nuclear receptor subfamily 1, group H, member 4 |
| PPARA | peroxisome proliferator-activated receptor alpha |
| PPARD | peroxisome proliferator-activated receptor delta |
| ADNP | activity-dependent neuroprotector homeobox |
| ESR2 | estrogen receptor 2 (ER beta) |
| ESRRB | estrogen-related receptor beta |
| MBTPS1 | membrane-bound transcription factor peptidase, site 1 |
| NR1I2 | nuclear receptor subfamily 1, group I, member 2 |
| NR2F2 | nuclear receptor subfamily 2, group F, member 2 |
| NR3C1 | nuclear receptor subfamily 3, group C, member 1 (glucocorticoid receptor) |
| PGR | progesterone receptor |
| TFCP2L1 | transcription factor CP2-like 1 |
